# Supplementary material for: Impact of Soy β-Conglycinin Peptides on PCSK9 Protein Expression in HepG2 Cells
Source: Nutrients. 2021 Dec 31;14(1):193. doi: 10.3390/nu14010193 (PMC8747205; doi:10.3390/nu14010193)
Supplement: Supplementary file 1 [file nutrients-14-00193-s001.zip › nutrients-1500163-supplementary-done.pdf]

nutrients

Supplemental Figure S1. β-Galactosidase activity. It was assayed to assess treatment interference with transfection and/or protein expression. pCMV-β vector, encoding for β-galactosidase , was transfected in HepG2 cells.

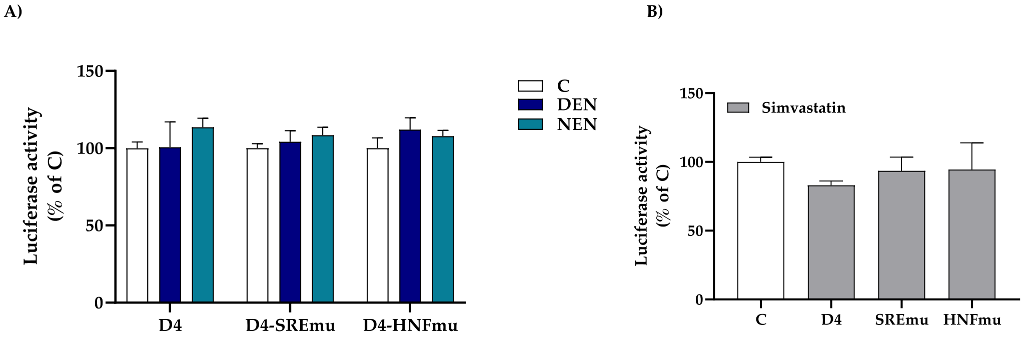

Panel A) shows luciferase activity upon treatment with YVVNPDN**DEN** (350 μM) and YVVNPDN**NEN** (350 μM) peptides. Panel B) shows luciferase activity upon treatment with simvastatin (20 μM). pCMV-β vector encoding for β-galactosidase was transfected in HepG2 cells. Cells were transiently transfected with pGL3-PCSK9-D4 plasmids (wild-type, SRE-mu and HNF-1-mu). SRE-mu, mutation in sterol regulatory element motif; HNF-mu, mutation in hepatocyte nuclear factor alpha motif; DEN, YVVNPDN**DEN**; NEN, YVVNPDN**NEN**

Supplemental Figure S2. PCSK9-LDLR binding activity. YVVNPDN**DEN** and YVVNPDN**NEN** peptides did not inhibit the PCSK9-LDLR binding.

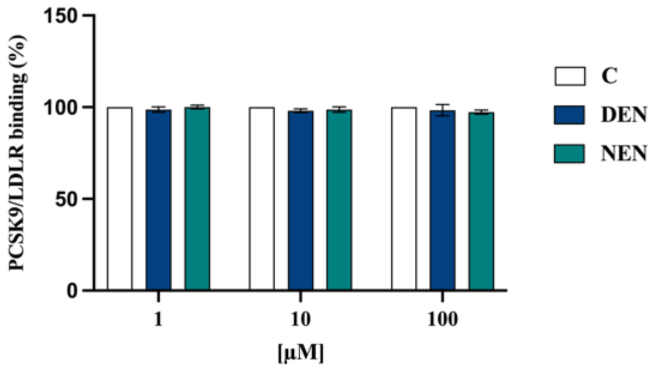

The YVVNPDN**DEN** and YVVNPDN**NEN** peptides were added to proper amount of His-tagged PCSK9 in each coated well with LDLR-AB domain. The percentage of PCSK9-LDLR binding inhibition was measured from the absorbance reduction at 450 nm. DEN, YVVNPDN**DEN**; NEN, YVVNPDN**NEN**;
